# Inactive status is an independent predictor of liver transplant waitlist mortality and is associated with a transplant centers median meld at transplant

**DOI:** 10.1371/journal.pone.0260000

**Published:** 2021-11-18

**Authors:** Jonathan Merola, Geliang Gan, Darren Stewart, Samantha Noreen, David Mulligan, Ramesh Batra, Danielle Haakinson, Yanhong Deng, Sanjay Kulkarni

**Affiliations:** 1 Department of Surgery, Division of Organ Transplantation, Yale School of Medicine, New Haven, Connecticut, United States of America; 2 Yale Center for Analytical Sciences, Yale School of Public Health, New Haven, Connecticut, United States of America; 3 United Network for Organ Sharing, Richmond, Virginia, United States of America; Imperial College Healthcare NHS Trust, UNITED KINGDOM

## Abstract

**Background:**

Approximately 30% of patients on the liver transplant waitlist experience at least one inactive status change which makes them temporarily ineligible to receive a deceased donor transplant. We hypothesized that inactive status would be associated with higher mortality which may differ on a transplant centers’ or donor service areas’ (DSA) Median MELD at Transplant (MMaT).

**Methods:**

Multi-state models were constructed (OPTN database;06/18/2013-06/08/2018) using DSA-level and transplant center-level data where MMaT were numerically ranked and categorized into tertiles. Hazards ratios were calculated between DSA and transplant center tertiles, stratified by MELD score, to determine differences in inactive to active transition probabilities.

**Results:**

7,625 (30.2% of sample registrants;25,216 total) experienced at least one inactive status change in the DSA-level cohort and 7,623 experienced at least one inactive status change in the transplant-center level cohort (30.2% of sample registrants;25,211 total). Inactive patients with MELD≤34 had a higher probability of becoming re-activated if they were waitlisted in a low or medium MMaT transplant center or DSA. Transplant rates were higher and lower re-activation probability was associated with higher mortality for the MELD 26–34 group in the high MMaT tertile. There were no significant differences in re-activation, transplant probability, or waitlist mortality for inactivated patients with MELD≥35 regardless of a DSA’s or center’s MMaT.

**Conclusion:**

This study shows that an inactive status change is independently associated with waitlist mortality. This association differs by a centers’ and a DSAs’ MMaT. Prioritization through care coordination to resolve issues of inactivity is fundamental to improving access.

## Introduction

Reducing liver transplant waitlist mortality is a priority for the transplant community and has driven recent changes in the allocation system where the sickest patients are afforded the greatest opportunity for transplantation [1, 2]. When determining waitlist mortality at a national, regional, donor service area (DSA), or transplant center-level, both active and inactive patients are used in the calculation [3]. Transplant rate also includes inactive patients, yet because they don’t receive organ offers, this important performance measure is unable to adjust for differences in waitlists between centers. Two centers may have similar rates of transplant and still have very different proportions of inactive patients on their waitlist.

Inactive status change has been shown to be an independent predictor of waitlist mortality in kidney transplant candidates [4]. To date, there has yet to be a comprehensive analysis of the impact of an inactive status change for liver transplant candidates. A quantitative determination of the impact of an inactive status change would improve transparency with patients and their providers, hopefully improve care coordination for waitlisted patients to resolve issues of inactivity, and potentially provide new insights into how transplant centers manage their waitlists.

There are several reasons why a patient may be made inactive on the waitlist, including but not limited to: incomplete testing, psychosocial issues, medical reasons, or the lack of financial clearance [5]. The challenge of comprehensively studying the impact of an inactive status change on waitlist outcomes stems from the dynamic nature of status changes. Ideally, one would want to measure the probability of a patient becoming inactive, measure the future probability of becoming active, determine their mortality in either active or inactive status, and measure transplant rate following activation. Conventional competing risks analysis can only model active patients to determine the probability of death versus transplant versus waitlist removal. Inactive patients are excluded in these analyses as inactive patients cannot "compete" for the transplant outcome [4].

Multi-state modeling uses a nested competing risk approach to dynamically measure an individuals’ probability of moving between active and inactive status, while simultaneously determining waitlist outcomes [6, 7]. With this methodology, we take a comprehensive look at inactive patients on the liver transplant waitlist and offer new insights into the effect of this status change on mortality and the future probability of obtaining a transplant. As geographical variation in waitlist mortality and transplant rate have been points of emphasis in the transplant community, we designed our study to measure differences in outcomes at both the DSA and transplant center-level grouped by their respective Median Meld at Transplant (MMaT). Given that approximately a third of all listed patients experience at least one inactive status change, the impact on this population of liver patients should provide insights that further our knowledge on waitlist outcomes.

## Materials and methods

The Yale University Human Investigation Committee approved the study protocol. This study was exempt from patient consent due to the use of de-identified data. This study used data from the Organ Procurement and Transplantation Network (OPTN). The OPTN data system includes donors, wait-listed candidates, and transplant recipients in the United States, submitted by members of the OPTN. The Health Resources and Services Administration of the U.S. Department of Health and Human Services provides oversight of activities of the OPTN contractor. All relevant data are fully available without restriction at the Scientific Registry of Transplant Recipients database (https://optn.transplant.hrsa.gov/data/request-data/). The authors did not have special access to this database which is publicly accessible to any researcher to reproduce the same analyses performed in this submission.

We constructed a semi-parametric, multi-state model using OPTN liver waitlist data from June 18, 2013 (initiation of Share 35 policy) to June 8, 2018. All patients included in the dataset where required to have a minimum of 1-year follow-up. Patients who never experienced an inactive status change, pediatric patients, those initially or subsequently listed with a MELD exception, Status 1 patients, individuals listed for multiple organs except those co-listed for kidney, and patients transplanted with deceased donors from outside the U.S. were excluded. MMaT was calculated for all patients (active and inactive) by DSA and transplant center, ranked, and then categorized into tertiles (low, medium, and high MMaT). Patients were stratified into the following MELD groups based on their MELD score at the time of their first inactive status change: ≤14, 15–25, 26–34, ≥35.

**Fig 1:**
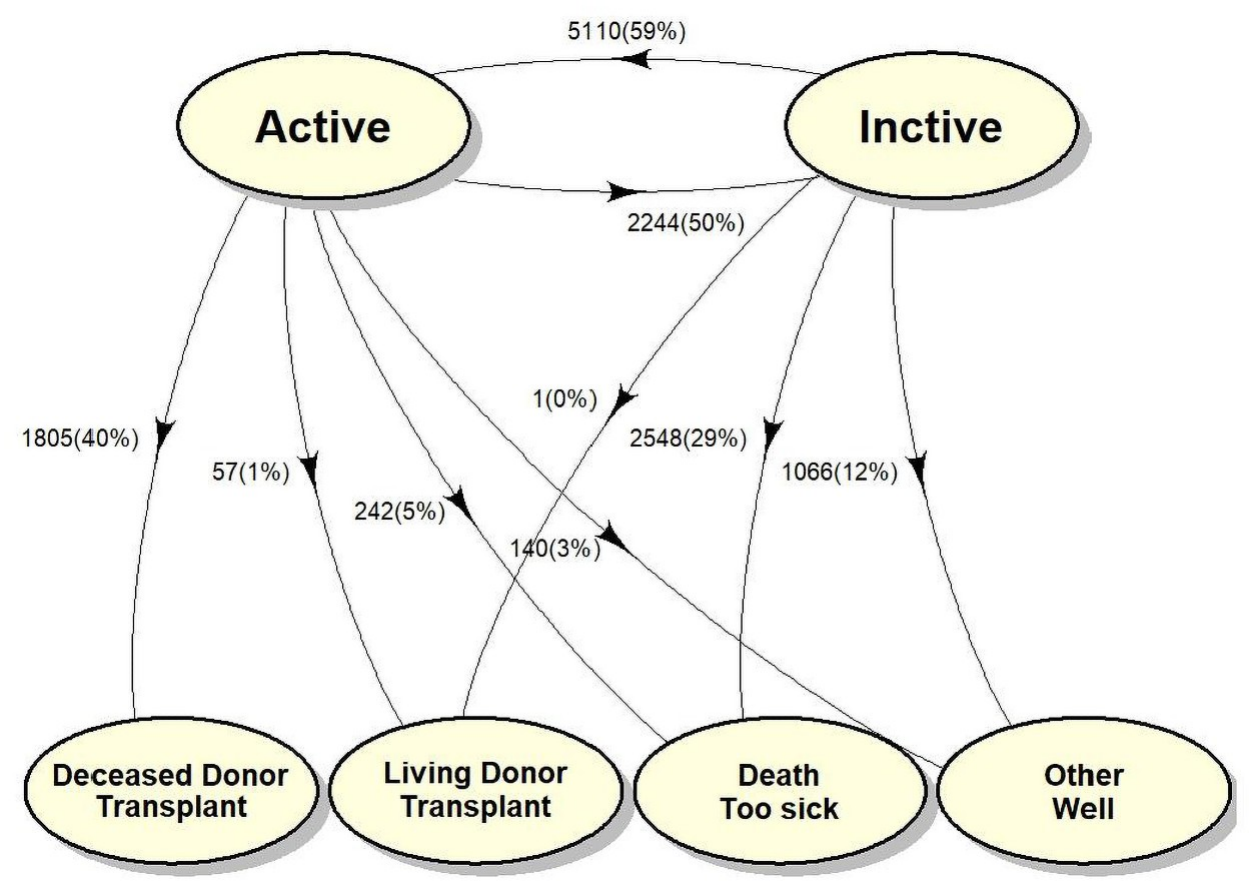
Multistate model schematic of transitions with competing risks outcomes. Inactive patients have no direct path to transplant. However, the inactive to active status transition allows the calculation of waitlist outcomes from active status. Frequencies and percentages of each transition provided next to each transition.

To assess the difference in outcomes at both the DSA and center-level, MELD groups defined at DSA and transplant center-level were entered into two separate multi-state models which allowed for the following nine transitions (Fig 1): Active to inactive status, active to deceased donor transplant, active to living donor transplant, active to death, active to well/other, inactive to active, inactive to living donor transplant, inactive to death and inactive to well/other. Patients entered the model when they experienced their first inactive status change. Inactive to deceased donor transition was not modelled as inactive patients cannot receive organ offers. However, the model allowed for the transition from inactive to active, from which the competing risk probability to deceased donor transplant, living donor transplant, death, or too well/other could be measured (Fig 1). The effect of risk factors on transition rates were modeled using Cox’s proportional hazards model for each of the transition hazards separately and was reported using hazard ratios (HR) as described forthwith. Each transition-specific model was adjusted for the following patient-level covariates: age at first inactive status change, diagnosis, race/ethnicity, blood type, insurance status, duration between listing and first inactive status change, MELD score at first inactive status change, and MELD at transplant. The proportional hazards assumption was evaluated by the scaled Schoenfeld residuals against time [8]. The model building process started with the null model which was built with the regression coefficients for all risk factors constrained to be identical for all 9 transitions. Each covariate was then sequentially allowed to differ across transitions by creating transition-specific covariates, while the other covariates were kept identical across transitions. The likelihood-ratio test was used to test the null hypothesis that the regression coefficients were identical for all transitions. The Akaike information criterion was used to select the best fit model. Based on the cumulative hazards estimated from the transition-specific Cox model on the transition hazards between states, the transition probabilities were estimated using Aalen-Johnsen estimators [9].

Demographic data was reported with means and SD for continuous variables and percentage and frequencies were used for categorical data. Statistical significance was set at the 0.05 level. Data management was done in SAS 9.4 (SAS Institute Inc., Cary, NC, USA) and the multistate modeling was done using R mstate package (version 3.1.0, R Core Team (2020) [10]; R: A language and environment for statistical computing, R Foundation for Statistical Computing, Vienna, Austria. URL https://www.r-project.org/).

## Results

### Study population and DSA/transplant center tertiles

S1 Table shows population demographics at the DSA-level for patients experiencing at least one inactive status change (n = 7,625; 30.2% of sample registrants, total 25,216). The MMaT was calculated per DSA, ranked, and then tertiles were determined with the following number of DSAs: low MMaT tertile (n = 17), medium MMaT tertile (n = 20), and high MMaT tertile (n = 15). The low tertile had a MMaT range of 18–25 (16.4% of patients), those in the middle tertile ranged from 26–29 (48.4%), and those in the highest tertile had MMaT ranging from 30–38 (35.2%). Low and medium MMaT DSAs had wide geographic distribution; however, since only 17% of DSAs are located in the Western U.S., it is noteworthy that 43.7% of high MMaT DSAs were located in this area. Similar distributions were noted in terms of age, blood type, liver disease diagnoses, education level, MELD score at inactivation, and primary insurance type across MMaT DSA tertiles. However, there was a notably higher percentage of A blood type patients in the low MMaT DSA group, a higher proportion of men in the medium MMaT DSA group, and higher percentage of Hispanics in the high MMaT DSA group.

S2 Table shows population demographics at the transplant center-level MMaT stratification for patients experiencing at least one inactive status change (n = 7,623; 30.2% of sample registrants, total 25,211). The difference in sample sizes between DSA and transplant center-level data was due to the observation that 5 centers had only one patient during the study period and those patients did not receive transplants resulting in their exclusion. The MMaT was calculated per transplant center, ranked, and tertiles were determined with the following number of centers: low MMaT tertile (n = 38), medium MMaT tertile (n = 41), and high MMaT tertile (n = 40). The lowest tertile included MMaT scores from 9–25 (28.4% of patients), the middle tertile ranged from 26–31 (40.7%), and those in the highest tertile MMaT ranged from 32–39 (30.9%). Low and medium MMaT transplant programs had wide geographic distribution; however, as with DSA-level stratification, we noted a markedly disproportionate representation (40.0%) of high MMaT transplant centers in the Western U.S. Similar demographic distributions were noted in the transplant center-level data, although there was greater similarity in blood type and gender across MMaT groups and the higher percentage of Hispanics in the high MMaT group remained. The most common reasons for the first inactive status change in both the DSA and transplant center-level data included patients becoming too ill for transplantation (53.0%), incomplete candidate workup (12.3%), and issues with insurance coverage (11.3%) (See Tables 1 and 2 for complete set of inactive status change reasons).

**Table 1:** 

	*Transplant DSA-Level*			*Total (N = 7482)*
	*low MELD DSA (N = 1231)*	*medium MELD DSA (N = 3671)*	*high MELD DSA (N = 2580)*	
**Reason for Inactive Status**				
Candidate cannot be contacted	0011 (00.89%)	0050 (01.36%)	0043 (01.67%)	0104 (01.39%)
Candidate choice	0070 (05.69%)	0204 (05.56%)	0099 (03.84%)	0373 (04.99%)
Living donor transplant only	0000 (00.00%)	0001 (00.03%)	0000 (00.00%)	0001 (00.01%)
Candidate work-up incomplete	0131 (10.64%)	0494 (13.46%)	0292 (11.32%)	0917 (12.26%)
Inappropriate substance use	0037 (03.01%)	0171 (04.66%)	0190 (07.36%)	0398 (05.32%)
Insurance issues	0143 (11.62%)	0409 (11.14%)	0293 (11.36%)	0845 (11.29%)
Medical non-compliance	0049 (03.98%)	0172 (04.69%)	0100 (03.88%)	0321 (04.29%)
Physician/Surgeon unavailable	0003 (00.24%)	0001 (00.03%)	0010 (00.39%)	0014 (00.19%)
TX Pending	0000 (00.00%)	0006 (00.16%)	0017 (00.66%)	0023 (00.31%)
Removal pending UNET data correction	0001 (00.08%)	0004 (00.11%)	0003 (00.12%)	0008 (00.11%)
Temporarily too sick	0737 (59.87%)	2016 (54.92%)	1214 (47.05%)	3967 (53.02%)
Temporarily too well	0039 (03.17%)	0109 (02.97%)	0298 (11.55%)	0446 (05.96%)
Weight currently inappropriate	0010 (00.81%)	0034 (00.93%)	0021 (00.81%)	0065 (00.87%)

**Table 2:** 

	*Transplant Center-Level*			*Total (N = 7480)*
	*Low MMaT center (N = 2140)*	*Medium MMaT center (N = 3078)*	*High MMaT center (N = 2262)*	
**Reason for Inactive Status**				
Candidate cannot be contacted	0025 (01.17%)	0048 (01.56%)	0031 (01.37%)	0104 (01.39%)
Candidate choice	0147 (06.87%)	0141 (04.58%)	0085 (03.76%)	0373 (04.99%)
Living donor transplant only	0001 (00.05%)	0000 (00.00%)	0000 (00.00%)	0001 (00.01%)
Candidate work-up incomplete	317 (14.81%)	331 (10.75%)	269 (11.89%)	917 (12.26%)
Inappropriate substance use	0080 (03.74%)	0159 (05.17%)	0159 (07.03%)	0398 (05.32%)
Insurance issues	228 (10.65%)	375 (12.18%)	241 (10.65%)	844 (11.28%)
Medical non-compliance	0110 (05.14%)	0122 (03.96%)	0089 (03.93%)	0321 (04.29%)
Physician/Surgeon unavailable	0000 (00.00%)	0014 (00.45%)	0000 (00.00%)	0014 (00.19%)
TX Pending	0001 (00.05%)	0012 (00.39%)	0010 (00.44%)	0023 (00.31%)
Removal pending UNET data correction	0001 (00.05%)	0004 (00.13%)	0003 (00.13%)	0008 (00.11%)
Temporarily too sick	1147 (53.60%)	1719 (55.85%)	1100 (48.63%)	3966 (53.02%)
Temporarily too well	0070 (03.27%)	0122 (03.96%)	0254 (11.23%)	0446 (05.96%)
Weight currently inappropriate	0013 (00.61%)	0031 (01.01%)	0021 (00.93%)	0065 (00.87%)

### Inactive to active status transitions stratified by DSA and transplant center MMaT tertile

For patients with MELD scores of 34 or less, the chances of converting from inactive to active status were approximately 2-fold to 2.5-fold higher if they were listed in a DSA with a low or medium MMaT compared to a high MMaT DSA (Table 3; p-values <0.001). We noted that the effect sizes were largest in the MELD 15–25 group (Low vs. High MMaT tertile, HR 2.26 [1.97, 2.60]; Medium vs. High MMaT tertile, HR 2.13 [1.90, 2.39]). There was no statistically significant difference in resolution of waitlist inactivity noted across MMaT DSA tertiles for patients with MELD scores ≥35.

**Table 3:** Hazard ratios of transition from inactive to active status stratified by DSA MMaT tertile.

		Inactive to Active	
MELD at Inactive Status Change	DSA MMaT Tertile Comparisons	Hazard Ratio	*P* value
MELD ≤ 14	Low vs. Medium DSA	0.85 (0.68, 1.05)	0.135
	Low vs. High DSA	2.12 (1.68, 2.69)	< .001
	Medium vs. High DSA	2.50 (2.11, 2.98)	< .001
MELD 15–25	Low vs. Medium DSA	1.06 (0.95, 1.19)	0.304
	Low vs. High DSA	2.26 (1.97, 2.59)	< .001
	Medium vs. High DSA	2.13 (1.90, 2.39)	< .001
MELD 26–34	Low vs. Medium DSA	1.05 (0.84, 1.30)	0.671
	Low vs. High DSA	2.09 (1.64, 2.66)	< .001
	Medium vs. High DSA	1.99 (1.66, 2.39)	< .001
MELD ≥ 35	Low vs. Medium DSA	0.87 (0.58, 1.28)	0.471
	Low vs. High DSA	0.97 (0.66, 1.43)	0.875
	Medium vs. High DSA	1.12 (0.89, 1.41)	0.337

Geographic disparities in resolution of inactivity across DSAs may reflect differing practice patterns or different patient factors. To better elucidate the association of MMaT with the likelihood of transitioning from inactive to active status, we analyzed differences by MMaT tertiles calculated at the individual transplant center-level (Table 4). Similar to our analysis using DSA-level data, we noted statistically significant differences in inactive to active transitions for patients with MELD scores ≤34 for patients listed at transplant centers with a low or medium MMaT. Different from the DSA-level comparisons, low MMaT tertile transplant centers had a statistically higher likelihood of activating their patients compared to medium MMaT centers (HR 1.43 [1.20, 1.70]). For patients with a MELD score ≥35 there was no statistically significant difference in inactive to active transitions across MMaT tertiles.

**Table 4:** Hazard ratios of transition from inactive to active status stratified by transplant center MMaT tertile.

		Inactive to active	
MELD at Inactive Status Change	MMaT Tertile Comparisons	Hazard Ratio	P value
MELD ≤14	Low vs. Medium Center	1.43 (1.20, 1.70)	< .001
	Low vs. High Center	2.46 (2.02, 2.20)	< .001
	Medium vs. High Center	1.72 (1.42, 2.09)	< .001
MELD 15–25	Low vs. Medium Center	1.19 (1.08, 1.32)	< .001
	Low vs. High Center	2.09 (1.85, 2.37)	< .001
	Medium vs. High Center	1.76 (1.56, 1.98)	< .001
MELD 26–34	Low vs. Medium Center	1.26 (1.04, 1.52)	0.017
	Low vs. High Center	1.84 (1.50, 2.26)	< .001
	Medium vs. High Center	1.46 (1.21, 1.77)	< .001
MELD ≥35	Low vs. Medium Center	0.90 (0.70, 1.22)	0.477
	Low vs. High Center	1.01 (0.75, 1.36)	0.934
	Medium vs. High Center	1.13 (0.88, 1.45)	0.328

### Deceased donor transplant probability stratified by DSA and transplant center MMaT tertile

**Fig 2:**
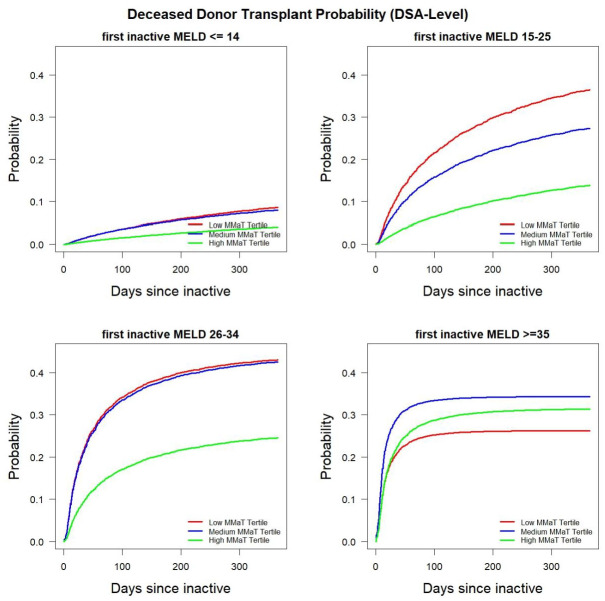
Probability of receiving deceased donor transplant among inactive waitlist candidates stratified by candidate MELD group and MMaT at the DSA-level.

**Fig 3:**
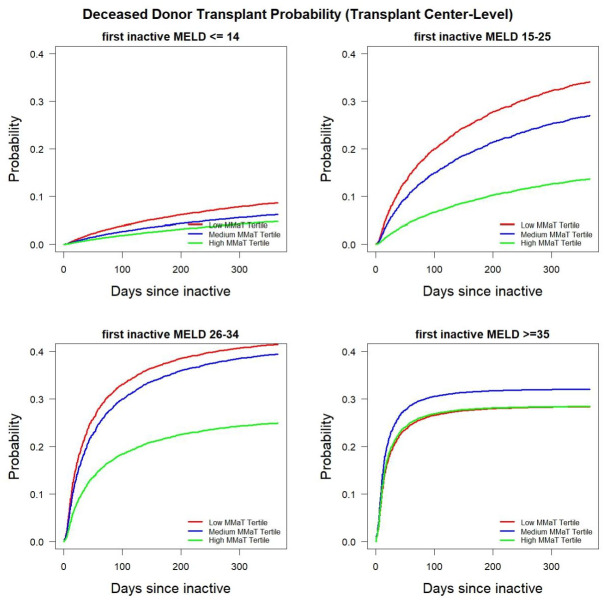
Probability of receiving deceased donor transplant among inactive waitlist candidates stratified by candidate MELD group and MMaT at the transplant center-level.

Given the statistically significant differences in inactive to active status transitions in lower MMaT DSA’s and centers, we used multi-state modeling to determine if this finding translated into differences in the probability of obtaining a deceased donor transplant for inactive patients. Fig 2 shows the probability of being transplanted in different MELD groups. For patients with MELD scores in either the 15–25 or 26–34 range, there was a higher probability of inactive patients resolving issues of inactivity and subsequently receiving a deceased donor liver transplant in DSA’s with a low or medium MMaT. In patients with MELD scores ≥ 35 there was a modest advantage if a patient was in a medium MMaT DSA. It should be reinforced that our dataset started at the implementation of Share 35 and it is noteworthy that the probabilities of deceased donor transplant are similar between DSAs. To confirm these finding, we performed the same analysis on transplant center-level data. Fig 3 shows transplant centers with a low or medium MMaT have a higher likelihood of transplanting inactive patients with MELD scores between 15–34. Again, only a modest effect size difference were noted in the MELD ≥ 35 group, confirming our DSA-level observation regarding the possible impact of Share 35 also seen at the transplant center-level.

### Probability of waitlist mortality stratified by DSA and transplant center MMaT tertile

**Fig 4:**
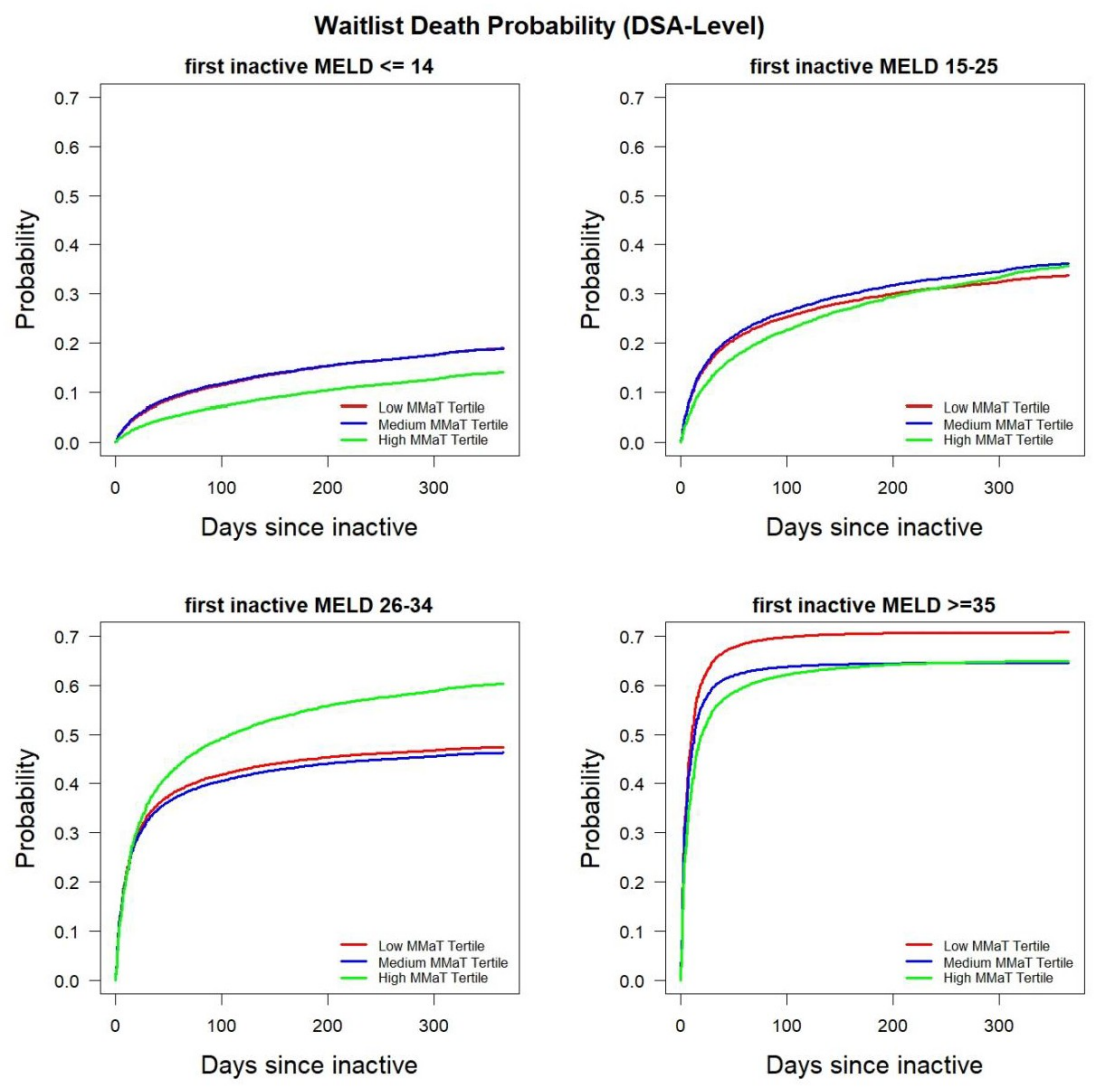
Probability of death among inactive waitlist candidates stratified by candidate MELD group and MMaT at the transplant center-level.

**Fig 5:**
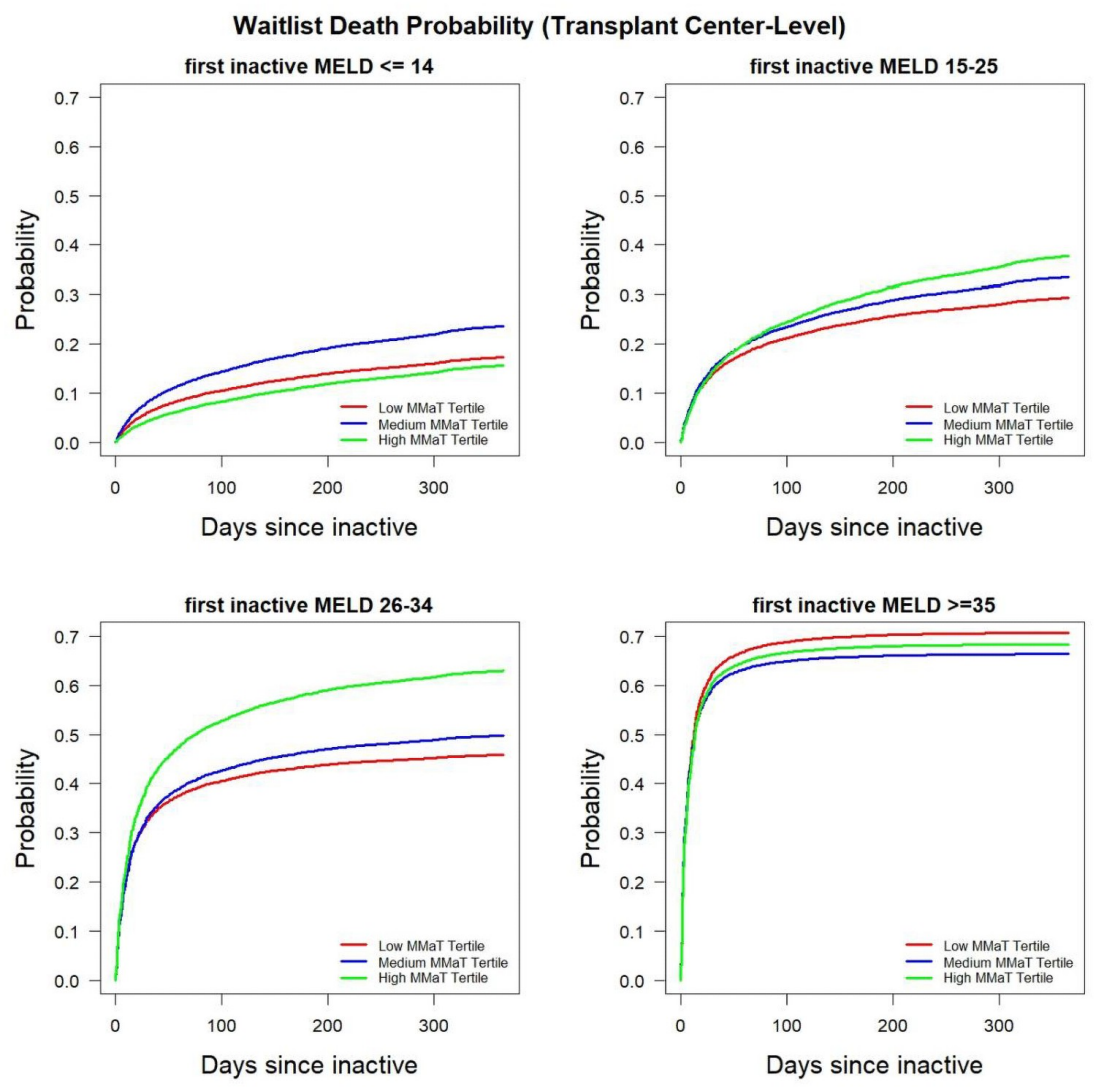
Probability of death among inactive waitlist candidates stratified by candidate MELD group and MMaT at the DSA-level.

Using multi-state models, we also determined waitlist mortality at the DSA and transplant center-level. Because of the ability to adjust for activity status, the model provides a cumulative probability of waitlist mortality from inactive patients transitioning to death and inactive patients transitioning to active status who ultimately die. Figs 4 and 5 show the probability of death stratified by DSA and transplant center MMaT tertile. Both plots show the greatest impact of death for MELD 26–34 patients who were waitlisted in a high MMaT DSA or high MMaT transplant centers. We noted that there were differences in MELD≥35 patients with those in low MMaT DSA or transplant centers having a higher mortality; however, the effect size was small. Waitlist mortality in the MELD≥35 group noted at the DSA and transplant-center level confirm findings we noted in the deceased donor transplant probability, which are consistent with the aims of Share 35.

## Discussion

This study demonstrates significant differences between transplant centers on resolving issues of inactivity on the liver transplant waitlist. The higher the MMaT of a DSA or transplant center, the less likely patients with MELD scores <35 were re-activated after being placed into inactive status. This finding is likely a contributory factor to the substantially higher waitlist mortality for inactive patients within the MELD 15–25 and 26–34 categories listed in DSAs or centers within the highest MMaT tertile. Patients who were made inactive and had MELD scores ≥ 35 had a similar likelihood of obtaining a transplant regardless of their DSA or centers’ MMaT, suggesting broader distribution of livers with Share 35 policy has improved access to transplant for these most severely ill patients. Our findings suggest that discordant waitlist management practices may, in part, perpetuate disparities in access to liver transplantation. They also show that transplant centers with greater access to liver grafts, presumably those within a lower MMaT DSA, emphasize activating high MELD patients and doing so to the same degree as centers within a high MMaT DSAs.

Liver allocation policy strives to achieve goals set forth by the U.S. Health and Human Services’ Final Rule, aiming to decrease waitlist mortality and reduce geographic disparities in transplant access, among other important goals [1]. Recent liver allocation policy changes focus on reducing geographic disparity by enabling broader sharing of organs [2, 11]. Equity in transplantation under the new liver allocation model assumes similar practice patterns across transplant centers, including organ acceptance practices and patient readiness for transplant. Although a transplant center may not be able to address all causes of inactivity, it is clear that patients with less access to healthcare, social support, or financial resources would have greater difficulty resolving issues of inactivity [12]. Our analysis does confirm that in groups with the highest medical complexity (MELD>35), there is equity in inactive to active transitions across MMaT groups of DSA’s and transplant centers. Therefore, if the focus is on providing transplants for inactive patients, this goal appears achievable. In this context, the resolution of inactivity issues requires care coordination and a multifaceted approach to waitlist management that should be translatable regardless of a patient’s MELD score or access to liver grafts.

The question arises if transplant centers in areas of the country with greater access to organs understand that activating lower MELD patients will allow them to obtain a transplant. Alternatively, transplant centers with less access to organs focus less on inactive patients with lower MELD scores because of the inability to obtain suitable organ offers. However, regardless of the level of access to liver grafts in a particular geographic area, addressing inactivity issues is essential to provide patients with an opportunity to obtain a deceased donor liver transplant offer. Failure to resolve inactivity issues will understandably result in higher waitlist mortality, which has been shown in studies on patients waiting for kidney transplants and demonstrated in this study for waitlisted liver patients [4]. Presumably, broader sharing will improve access to liver grafts for centers in high MMaT groups and it will be essential in future analyses to determine if this latest policy will result in changes in transplant center waitlist management practices with a greater focus on activating their inactive patients, regardless of their MELD score.

This study has the following limitations. It is based on the retrospective OPTN database, and thus, robust causal inference is difficult to establish. This database only includes information on the U.S. liver transplant waitlist, where issues related to inactive status, such as insurance issues, are likely different internationally. Thus, the generalizability of this analysis to non-U.S. transplant programs is limited. We use the probability of inactive to active status transition as a surrogate marker of waitlist management. The clinical practice of liver patients is complex, multimodal, and encompasses several aspects of care delivery that cannot be collapsed into a single probability estimate. Furthermore, there are several other factors that transplant centers do not control, including referral practices, distance from a transplant center, and changing demographics of patients. However, once a patient is placed on the waitlist the transplant center has an ethical and fiduciary responsibility to provide the best opportunity to receive a transplant, which can only be achieved in active status. Lastly, we did not perform this analysis under the current allocation system implemented on February 4, 2020. There is insufficient data to conduct such an analysis, and our model requires at least 1-year follow-up from the date of listing. When sufficient data is available, comparisons between the new broader sharing policy and prior allocation system should provide important insights into the relationship of changing waitlist management practices when access to liver grafts is improved.

## Conclusions

Our study shows significant differences in how successful transplant centers are in resolving inactivity issues and how this metric is associated with a centers’ MMaT. Establishing equity in healthcare mandates equal opportunity for life-saving treatments regardless of social determinants [13]. As inactive status removes the opportunity for life-saving treatment, the starting point in establishing equity in liver transplant access is first to confer active status, which is best conducted under the coordination, guidance, and management of transplant centers.

## Supporting information

S1 Table Study population demographics for patients experiencing at least one inactive status change (DSA-level). (DOCX)

S2 Table Study population demographics for patients experiencing at least one inactive status change (transplant center-level). (DOCX)

S3 Table Re-activation rates for the most common reasons for inactivity. (DOCX)

S4 Table Re-activation rates for the most common reasons for inactivity (transplant center-level). (DOCX)

S5 Table Association of number of days in inactive status and waitlist death. (DOCX)

S6 Table Detailed patient demographics of individuals experiencing inactivity during the follow-up period. (DOCX)

## Abbreviations

DSA: Donor Service Area
MELD: Model for End-Stage Liver Disease
MMaT: Median MELD at Transplant
OPTN: Organ Procurement and Transplantation Network
