# Supplementary material for: Inactive status is an independent predictor of liver transplant waitlist mortality and is associated with a transplant centers median meld at transplant
Source: PLoS One. 2021 Nov 18;16(11):e0260000. doi: 10.1371/journal.pone.0260000 (PMC8601542; doi:10.1371/journal.pone.0260000)
Supplement: S1 Table — (DOCX) [file pone.0260000.s001.docx]

**Supplementary Table 1. Study Population Demographics for Patients Experiencing at Least One Inactive Status Change (DSA-Level)**

|  | ***Transplant DSA*** | | |  |
| --- | --- | --- | --- | --- |
|  | ***Low MMaT DSA (N = 1248)*** | ***Medium MMaT DSA (N = 3696)*** | ***High MMaT DSA (N = 2681)*** | ***Total (N = 7625)*** |
| **Recipient Age at Registration** | | | | |
| Mean (SD) | 55.33 (10.26) | 55.02 (9.96) | 54.25 (10.02) | 54.80 (10.04) |
| Median (IQR) | 57.0 (50.0 – 63.0) | 57.0 (50.0 – 62.0) | 56.0 (49.0 – 61.0) | 57.0 (50.0 – 62.0) |
| **Age at First Inactive Status Change** | | | | |
| Mean (SD) | 55.82 (10.25) | 55.60 (9.98) | 54.96 (10.02) | 55.41 (10.04) |
| Median (IQR) | 58.0 (50.5 – 63.1) | 57.2 (50.4 – 62.8) | 56.5 (49.4 – 62.1) | 57.1 (50.1 – 62.6) |
| **Time to First Inactive (months)** | | | | |
| Mean (SD) | 6.01 (8.40) | 7.02 (9.19) | 8.64 (11.25) | 7.43 (9.90) |
| Median (IQR) | 2.6 (0.5 – 8.4) | 3.2 (0.7 – 10.1) | 3.8 (0.3 – 13.0) | 3.2 (0.5 – 10.6) |
| **MELD at LISTING** | | | | |
| Mean (SD) | 19.89 (7.73) | 19.48 (7.92) | 20.15 (8.79) | 19.79 (8.21) |
| Median (IQR) | 18.0 (14.0 – 24.0) | 18.0 (14.0 – 23.0) | 18.0 (14.0 – 26.0) | 18.0 (14.0 – 24.0) |
| **MELD at First Inactive Status Change** | | | | |
| Mean (SD) | 21.73 (8.66) | 21.56 (9.03) | 22.71 (10.80) | 21.99 (9.65) |
| Median (IQR) | 20.0 (16.0 – 26.0) | 20.0 (15.0 – 27.0) | 21.0 (14.0 – 31.0) | 20.0 (15.0 – 28.0) |
| **MELD Change from Listing to First Inactive** | | | | |
| Mean (SD) | 1.84 (5.12) | 2.07 (5.53) | 2.56 (7.32) | 2.21 (6.17) |
| Median (IQR) | 0.0 (0.0 – 3.0) | 0.0 (0.0 – 4.0) | 0.0 (0.0 – 4.0) | 0.0 (0.0 – 4.0) |
| **Gender** | | | | |
| Female | 0546 (43.75%) | 1441 (38.99%) | 1155 (43.08%) | 3142 (41.21%) |
| Male | 0702 (56.25%) | 2255 (61.01%) | 1526 (56.92%) | 4483 (58.79%) |
| **Blood Type** | | | | |
| A | 0555 (44.47%) | 1496 (40.48%) | 1010 (37.67%) | 3061 (40.14%) |
| B | 0127 (10.18%) | 0381 (10.31%) | 0311 (11.60%) | 0819 (10.74%) |
| AB | 0027 (02.16%) | 0120 (03.25%) | 0113 (04.21%) | 0260 (03.41%) |
| O | 0539 (43.19%) | 1699 (45.97%) | 1247 (46.51%) | 3485 (45.70%) |
| **Primary diagnosis at registration** | | | | |
| Acute Hepatic Necrosis | 0019 (01.52%) | 0044 (01.19%) | 0054 (02.01%) | 0117 (01.53%) |
| Non-Cholestatic Cirrhosis | 1050 (84.13%) | 3160 (85.50%) | 2334 (87.06%) | 6544 (85.82%) |
| Cholestatic Liver Disease/Cirrhosis | 0094 (07.53%) | 0281 (07.60%) | 0166 (06.19%) | 0541 (07.10%) |
| Biliary Atresia | 0003 (00.24%) | 0006 (00.16%) | 0006 (00.22%) | 0015 (00.20%) |
| Metabolic Disease | 0024 (01.92%) | 0081 (02.19%) | 0031 (01.16%) | 0136 (01.78%) |
| Malignant Neoplasms | 0017 (01.36%) | 0052 (01.41%) | 0032 (01.19%) | 0101 (01.32%) |
| Benign Neoplasms | 0004 (00.32%) | 0005 (00.14%) | 0005 (00.19%) | 0014 (00.18%) |
| Other | 0037 (02.96%) | 0067 (01.81%) | 0053 (01.98%) | 0157 (02.06%) |
| **Race** | | | | |
| non-Hispanic White | 0996 (79.81%) | 2885 (78.06%) | 1739 (64.86%) | 5620 (73.70%) |
| non-Hispanic Black | 0122 (09.78%) | 0311 (08.41%) | 0153 (05.71%) | 0586 (07.69%) |
| Hispanic | 0087 (06.97%) | 0393 (10.63%) | 0587 (21.89%) | 1067 (13.99%) |
| Asian/Other | 0043 (03.45%) | 0107 (02.90%) | 0202 (07.53%) | 0352 (04.62%) |
| **General U.S. Regions** | | | | |
| Northeast | 0122 (09.78%) | 1359 (36.77%) | 0244 (09.10%) | 1725 (22.62%) |
| Southeast | 0615 (49.28%) | 1317 (35.63%) | 0588 (21.93%) | 2520 (33.05%) |
| Midwest | 0496 (39.74%) | 0894 (24.19%) | 0677 (25.25%) | 2067 (27.11%) |
| West | 0015 (01.20%) | 0126 (03.41%) | 1172 (43.72%) | 1313 (17.22%) |
| **Education Level** | | | | |
| No or Grade School Education | 0046 (03.90%) | 0157 (04.49%) | 0197 (07.77%) | 0400 (05.54%) |
| High School Graduate | 0821 (69.58%) | 2461 (70.31%) | 1721 (67.84%) | 5003 (69.32%) |
| College Degree or Higher | 0313 (26.53%) | 0882 (25.20%) | 0619 (24.40%) | 1814 (25.14%) |
| **Primary Insurance** | | | | |
| Public | 0594 (47.60%) | 1768 (47.84%) | 1288 (48.04%) | 3650 (47.87%) |
| Private | 0647 (51.84%) | 1887 (51.06%) | 1358 (50.65%) | 3892 (51.04%) |
| Other | 0007 (00.56%) | 0041 (01.11%) | 0035 (01.31%) | 0083 (01.09%) |
