# Supplementary material for: Inactive status is an independent predictor of liver transplant waitlist mortality and is associated with a transplant centers median meld at transplant
Source: PLoS One. 2021 Nov 18;16(11):e0260000. doi: 10.1371/journal.pone.0260000 (PMC8601542; doi:10.1371/journal.pone.0260000)
Supplement: S2 Table — (DOCX) [file pone.0260000.s002.docx]

**Supplementary Table 2. Study Population Demographics for Patients Experiencing at Least One Inactive Status Change (Transplant Center-Level)**

|  | ***Transplant Center*** | | |  |
| --- | --- | --- | --- | --- |
|  | ***Low MMaT center (N = 2166)*** | ***Medium MMaT center (N = 3099)*** | ***High MMaT center (N = 2358)*** | ***Total (N = 7623)*** |
| **Recipient Age at Registration** | | | | |
| Mean (SD) | 54.92 (10.38) | 54.90 (9.88) | 54.60 (9.90) | 54.81 (10.03) |
| Median (IQR) | 57.0 (50.0 – 62.0) | 57.0 (50.0 – 62.0) | 56.0 (49.0 – 62.0) | 57.0 (50.0 – 62.0) |
| **Age at First Inactive Status Change** | | | | |
| Mean (SD) | 55.41 (10.38) | 55.58 (9.87) | 55.21 (9.91) | 55.42 (10.03) |
| Median (IQR) | 57.2 (50.2 – 63.0) | 57.2 (50.4 – 62.7) | 57.0 (49.9 – 62.3) | 57.1 (50.1 – 62.6) |
| **Time to First Inactive (months)** | | | | |
| Mean (SD) | 6.04 (8.39) | 8.35 (10.59) | 7.50 (10.08) | 7.43 (9.90) |
| Median (IQR) | 2.6 (0.5 – 8.4) | 3.9 (0.7 – 12.0) | 3.1 (0.2 – 11.0) | 3.2 (0.5 – 10.6) |
| **MELD at LISTING** | | | | |
| Mean (SD) | 19.35 (7.78) | 19.44 (7.92) | 20.64 (8.88) | 19.78 (8.21) |
| Median (IQR) | 18.0 (14.0 – 23.0) | 18.0 (14.0 – 23.0) | 19.0 (14.0 – 26.0) | 18.0 (14.0 – 24.0) |
| **MELD at First Inactive Status Change** | | | | |
| Mean (SD) | 21.21 (8.67) | 21.54 (9.17) | 23.30 (10.89) | 21.99 (9.64) |
| Median (IQR) | 20.0 (15.0 – 26.0) | 20.0 (15.0 – 27.0) | 21.0 (14.0 – 32.0) | 20.0 (15.0 – 28.0) |
| **MELD Change from Listing to First Inactive** | | | | |
| Mean (SD) | 1.87 (5.06) | 2.09 (5.81) | 2.66 (7.40) | 2.21 (6.17) |
| Median (IQR) | 0.0 (0.0 – 3.0) | 0.0 (0.0 – 4.0) | 0.0 (0.0 – 5.0) | 0.0 (0.0 – 4.0) |
| **Gender** | | | | |
| Female | 0920 (42.47%) | 1229 (39.66%) | 0993 (42.11%) | 3142 (41.22%) |
| Male | 1246 (57.53%) | 1870 (60.34%) | 1365 (57.89%) | 4481 (58.78%) |
| **Blood Type** | | | | |
| A | 0885 (40.86%) | 1268 (40.92%) | 0907 (38.46%) | 3060 (40.14%) |
| B | 0233 (10.76%) | 0311 (10.04%) | 0275 (11.66%) | 0819 (10.74%) |
| AB | 0062 (02.86%) | 0093 (03.00%) | 0105 (04.45%) | 0260 (03.41%) |
| O | 0986 (45.52%) | 1427 (46.05%) | 1071 (45.42%) | 3484 (45.70%) |
| **Primary diagnosis at registration** | | | | |
| Acute Hepatic Necrosis | 0038 (01.75%) | 0035 (01.13%) | 0043 (01.82%) | 0116 (01.52%) |
| Non-Cholestatic Cirrhosis | 1806 (83.38%) | 2688 (86.74%) | 2050 (86.94%) | 6544 (85.85%) |
| Cholestatic Liver Disease/Cirrhosis | 0189 (08.73%) | 0203 (06.55%) | 0149 (06.32%) | 0541 (07.10%) |
| Biliary Atresia | 0004 (00.18%) | 0006 (00.19%) | 0005 (00.21%) | 0015 (00.20%) |
| Metabolic Disease | 0045 (02.08%) | 0072 (02.32%) | 0019 (00.81%) | 0136 (01.78%) |
| Malignant Neoplasms | 0027 (01.25%) | 0043 (01.39%) | 0031 (01.31%) | 0101 (01.32%) |
| Benign Neoplasms | 0005 (00.23%) | 0004 (00.13%) | 0005 (00.21%) | 0014 (00.18%) |
| Other | 0052 (02.40%) | 0048 (01.55%) | 0056 (02.37%) | 0156 (02.05%) |
| **Race** | | | | |
| non-Hispanic White | 1728 (79.78%) | 2375 (76.64%) | 1515 (64.25%) | 5618 (73.70%) |
| non-Hispanic Black | 0167 (07.71%) | 0275 (08.87%) | 0144 (06.11%) | 0586 (07.69%) |
| Hispanic | 0195 (09.00%) | 0356 (11.49%) | 0516 (21.88%) | 1067 (14.00%) |
| Asian/Other | 0076 (03.51%) | 0093 (03.00%) | 0183 (07.76%) | 0352 (04.62%) |
| **General U.S. Regions** | | | | |
| Northeast | 0643 (29.69%) | 0576 (18.59%) | 0505 (21.42%) | 1724 (22.62%) |
| Southeast | 0743 (34.30%) | 1377 (44.43%) | 0399 (16.92%) | 2519 (33.04%) |
| Midwest | 0701 (32.36%) | 1084 (34.98%) | 0282 (11.96%) | 2067 (27.12%) |
| West | 0079 (03.65%) | 0062 (02.00%) | 1172 (49.70%) | 1313 (17.22%) |
| **Education Level** | | | | |
| No or Grade School Education | 0096 (04.68%) | 0100 (03.40%) | 0204 (09.17%) | 0400 (05.54%) |
| High School Graduate | 1403 (68.34%) | 2074 (70.62%) | 1525 (68.54%) | 5002 (69.33%) |
| College Degree or Higher | 0554 (26.98%) | 0763 (25.98%) | 0496 (22.29%) | 1813 (25.13%) |
| **Primary Insurance** | | | | |
| Public | 0982 (45.34%) | 1491 (48.11%) | 1176 (49.87%) | 3649 (47.87%) |
| Private | 1167 (53.88%) | 1568 (50.60%) | 1156 (49.02%) | 3891 (51.04%) |
| Other | 0017 (00.78%) | 0040 (01.29%) | 0026 (01.10%) | 0083 (01.09%) |
