# Supplementary material for: Inactive status is an independent predictor of liver transplant waitlist mortality and is associated with a transplant centers median meld at transplant
Source: PLoS One. 2021 Nov 18;16(11):e0260000. doi: 10.1371/journal.pone.0260000 (PMC8601542; doi:10.1371/journal.pone.0260000)
Supplement: S3 Table — (DOCX) [file pone.0260000.s003.docx]

**Supplementary Table 3a. Re-activation Rates for the Most Common Reasons for Inactivity**

**(DSA-Level)**

|  | ***Re-activated after entering inactive state***  ***(DSA-Level)*** | |  |
| --- | --- | --- | --- |
|  | ***Yes (N = 3104)*** | ***No (N = 2625)*** | ***Total (N = 5729)*** |
| **Reason for Inactive Status** | | | |
| Candidate work-up incomplete | 0537 (58.56%) | 0380 (41.44%) | 0917 (16.01%) |
| Insurance issues | 0548 (64.85%) | 0297 (35.15%) | 0845 (14.75%) |
| Temporarily too sick | 2019 (50.89%) | 1948 (49.11%) | 3967 (69.24%) |
