# Supplementary material for: Inactive status is an independent predictor of liver transplant waitlist mortality and is associated with a transplant centers median meld at transplant
Source: PLoS One. 2021 Nov 18;16(11):e0260000. doi: 10.1371/journal.pone.0260000 (PMC8601542; doi:10.1371/journal.pone.0260000)
Supplement: S4 Table — (DOCX) [file pone.0260000.s004.docx]

**Supplementary Table 3b. Re-activation Rates for the Most Common Reasons for Inactivity**

**(Transplant Center-Level)**

|  | ***Re-activated after entering inactive state (Transplant Center-Level)*** | |  |
| --- | --- | --- | --- |
|  | ***Yes (N = 3103)*** | ***No (N = 2624)*** | ***Total (N = 5727)*** |
| **Reason for Inactive Status** | | | |
| Candidate work-up incomplete | 0537 (58.56%) | 0380 (41.44%) | 0917 (16.01%) |
| Insurance issues | 0547 (64.81%) | 0297 (35.19%) | 0844 (14.74%) |
| Temporarily too sick | 2019 (50.91%) | 1947 (49.09%) | 3966 (69.25%) |
