# Supplementary material for: Inactive status is an independent predictor of liver transplant waitlist mortality and is associated with a transplant centers median meld at transplant
Source: PLoS One. 2021 Nov 18;16(11):e0260000. doi: 10.1371/journal.pone.0260000 (PMC8601542; doi:10.1371/journal.pone.0260000)
Supplement: S5 Table — (DOCX) [file pone.0260000.s005.docx]

**Supplementary Table 4. Association of Number of Days in Inactive Status and Waitlist Death**

|  | ***Patient died in waiting list***  ***(DSA-Level)*** | |  | |
| --- | --- | --- | --- | --- |
|  | ***Yes (N = 2790)*** | ***No (N = 4835)*** | ***Total (N = 7625)*** | ***P Value*** |
| **Days in Inactive state** | | | | |
| Mean (SD) | 68.46 (136.38) | 178.43 (268.97) | 138.19 (235.54) | <0.001^***^ |
| Median (Range) | 17.0 (0.1 – 1350.9) | 63.0 (0.1 – 1795.9) | 37.8 (0.1 – 1795.9) |  |
