# Supplementary material for: Inactive status is an independent predictor of liver transplant waitlist mortality and is associated with a transplant centers median meld at transplant
Source: PLoS One. 2021 Nov 18;16(11):e0260000. doi: 10.1371/journal.pone.0260000 (PMC8601542; doi:10.1371/journal.pone.0260000)
Supplement: S6 Table — (DOCX) [file pone.0260000.s006.docx]

**Supplementary Table 5. Detailed Patient Demographics of Individuals Experiencing Inactivity During the Follow-up Period**

|  | ***Patient experiencing inactive state during follow-up (DSA-Level)*** | |  |
| --- | --- | --- | --- |
|  | ***Yes (N = 7625)*** | ***No (N = 17591)*** | ***Total (N = 25216)*** |
| **Recipient age at registration** | | | |
| N (N Missing) | 7625 (0) | 17591 (0) | 25216 (0) |
| Mean (IQR) | 54.80 (50.00 – 62.00) | 54.73 (49.00 – 62.00) | 54.75 (49.00 – 62.00) |
| Median (Range) | 57.0 (18.0 – 79.0) | 56.0 (18.0 – 79.0) | 56.0 (18.0 – 79.0) |
| **Recipient gender** | | | |
| Missing | 00000 (00.00%) | 00000 (00.00%) | 00000 (00.00%) |
| Female | 03142 (41.21%) | 06702 (38.10%) | 09844 (39.04%) |
| Male | 04483 (58.79%) | 10889 (61.90%) | 15372 (60.96%) |
| **Blood Type** | | | |
| Missing | 00000 (00.00%) | 00000 (00.00%) | 00000 (00.00%) |
| A | 03061 (40.14%) | 06485 (36.87%) | 09546 (37.86%) |
| B | 00819 (10.74%) | 02266 (12.88%) | 03085 (12.23%) |
| AB | 00260 (03.41%) | 00733 (04.17%) | 00993 (03.94%) |
| O | 03485 (45.70%) | 08107 (46.09%) | 11592 (45.97%) |
| **Primary diagnosis at registration** | | | |
| Missing | 00000 (00.00%) | 00002 (00.01%) | 00002 (00.01%) |
| Acute Hepatic Necrosis | 00117 (01.53%) | 00300 (01.71%) | 00417 (01.65%) |
| Non-Cholestatic Cirrhosis | 06544 (85.82%) | 14767 (83.96%) | 21311 (84.52%) |
| Cholestatic Liver Disease/Cirrhosis | 00541 (07.10%) | 01461 (08.31%) | 02002 (07.94%) |
| Biliary Atresia | 00015 (00.20%) | 00038 (00.22%) | 00053 (00.21%) |
| Metabolic Disease | 00136 (01.78%) | 00311 (01.77%) | 00447 (01.77%) |
| Malignant Neoplasms | 00101 (01.32%) | 00339 (01.93%) | 00440 (01.75%) |
| Benign Neoplasms | 00014 (00.18%) | 00051 (00.29%) | 00065 (00.26%) |
| Other | 00157 (02.06%) | 00322 (01.83%) | 00479 (01.90%) |
| **Recipient race** | | | |
| Missing | 00000 (00.00%) | 00000 (00.00%) | 00000 (00.00%) |
| non-Hispanic White | 05620 (73.70%) | 12494 (71.02%) | 18114 (71.84%) |
| non-Hispanic Black | 00586 (07.69%) | 01418 (08.06%) | 02004 (07.95%) |
| Hispanic | 01067 (13.99%) | 02900 (16.49%) | 03967 (15.73%) |
| Asian/Other | 00352 (04.62%) | 00779 (04.43%) | 01131 (04.49%) |
| **U.S. regions** | | | |
| Missing | 00000 (00.00%) | 00000 (00.00%) | 00000 (00.00%) |
| Northeast | 01725 (22.62%) | 04237 (24.09%) | 05962 (23.64%) |
| Southeast | 02520 (33.05%) | 06011 (34.17%) | 08531 (33.83%) |
| Midwest | 02067 (27.11%) | 03893 (22.13%) | 05960 (23.64%) |
| West | 01313 (17.22%) | 03450 (19.61%) | 04763 (18.89%) |
| **Recipient education level** | | | |
| Missing | 00408 (05.35%) | 00925 (05.26%) | 01333 (05.29%) |
| No or Grade School Education | 00400 (05.54%) | 01032 (06.19%) | 01432 (06.00%) |
| High School Graduate | 05003 (69.32%) | 11361 (68.17%) | 16364 (68.52%) |
| College Degree or Higher | 01814 (25.14%) | 04273 (25.64%) | 06087 (25.49%) |
| **Recipient primary source of payment** | | | |
| Missing | 00000 (00.00%) | 00002 (00.01%) | 00002 (00.01%) |
| Public | 03650 (47.87%) | 08016 (45.57%) | 11666 (46.27%) |
| Private | 03892 (51.04%) | 09354 (53.18%) | 13246 (52.53%) |
| Other | 00083 (01.09%) | 00219 (01.25%) | 00302 (01.20%) |
